# Characterization of a Novel *Bacillus glycinifermentans* Strain MGMM1 Based on Full Genome Analysis and Phenotypic Properties for Biotechnological Applications

**DOI:** 10.3390/microorganisms11061410

**Published:** 2023-05-26

**Authors:** Daniel Mawuena Afordoanyi, Roderic Gilles Claret Diabankana, Ernest Nailevich Komissarov, Evgenii Sergeyevich Kuchaev, Shamil Zavdatovich Validov

**Affiliations:** 1 Laboratory of Molecular Genetics and Microbiology Methods, Kazan Scientific Center of Russian Academy of Sciences, 420111 Kazan, Russiash.validov@knc.ru (S.Z.V.); 2 Tatar Scientific Research Institute of Agricultural Chemistry and Soil Science, FRC Kazan Scientific Center, Russian Academy of Sciences, 420111 Kazan, Russia

**Keywords:** *Bacillus glycinifermentans*, phenotypic properties, *Fusarium oxysporum*, biocontrol, full genome

## Abstract

*Bacillus* species have gained much attention based on their phenotypic characteristics and their genetic architecture as biological control agents and plant growth-promotor with bioremediation potential. In this study, we analyzed the whole genome of a novel strain, *Bacillus glycinifermentans* MGMM1, isolated from the rhizosphere of a weed plant (*Senna occidentalis*) and assayed its phenotypic characteristics, as well as antifungal and biocontrol ability. The whole genome analysis of MGMM1 identified 4259 putative coding sequences, with an encoding density of 95.75% attributed to biological functions, including genes involved in stimulating plant growth, such as acetolactate synthase, *als*S, and genes involved in the resistance to heavy metal antimony (*ars*B and *ars*C). AntiSMASH revealed the presence of biosynthetic gene clusters plipastatin, fengycin, laterocidine, geobacillin II, lichenysin, butirosin A and schizokinen. Tests in vitro confirmed that MGMM1 exhibited antifungal activity against *Fusarium oxysporum* f.sp. *radicis-lycopersici* (*Forl*) ZUM2407, *Alternaria alternata*, *F. graminearum* and *F.* spp. and produce protease, lipase amylase and cellulase. *Bacillus glycinifermentans* MGMM1 demonstrated proteolytic (4.82 ± 1.04 U/mL), amylolytic (0.84 ± 0.05 U/mL) and cellulosic (0.35 ± 0.02 U/mL) enzymatic activities, as well as indole-3-acetic acid production (48.96 ± 1.43 μg/mL). Moreover, the probiotic strain MGMM1 demonstrated a high biocontrol potential of inhibiting (up to 51.45 ± 8.08%) the development of tomato disease caused by *Forl* ZUM2407. These results suggest that *B. glycinifermentans* MGMM1 has significant potential as a biocontrol, plant growth-promoting agent in agriculture.

## 1. Introduction

Most attention has been given to bacilli for their antifungal properties, endophytic ability, plant hormone excretion and their ability to withstand adverse conditions due to their endospore formation [1,2,3,4]. This shows the robustness of *Bacillus* spp. and has therefore been grouped under the category of plant protectors [5]. The search for strong antifungal microbial agents against phytopathogens with a probiotic effect in farm animals is of great importance for replacing chemical base antimicrobials for safer agriculture products. The antifungal property of *Bacillus* spp. is mostly based on their small metabolites (lipopeptides), which include fengycin and surfactin [6]. Besides these lipopeptides, several enzymes also play a great role in the lysing of fungal cell walls, which includes β-glucanase, chitinase, protease, lipase and cellulase [7]. The enzymatic activity of *Bacillus* strains has garnered great interest due to their industrial applications [8]. The unique property of an individual *Bacillus* enzyme is based on their unique catalytic activity and stability of the enzymes through natural selection or directed evolution, which is of great interest to industries [9]. Some important enzymes include the thermostable α-amylase of *B. licheniformis*, the salt-tolerant protease of *B. subtilis*, the thermoalkalophilic lipase of *B. thermoleovorans* and the acidothermophilic cellulase of *B. subtilis* [10,11,12,13].

Strains of *Bacillus* species are also known to enhance nutrient uptake (solubilizing and mobilizing nutrients, such as phosphorus, nitrogen and potassium, and fixing atmospheric nitrogen), increase drought tolerance and alleviate negative salinity effects in the soil environment [14,15]. In addition, some *Bacillus* species have been reported to be effective in the degradation of pollutants, such as hydrocarbon waste and heavy metals [16]. For example, *Bacillus arsenicus* Con a/3T and *Bacillus indicus* strain Sd/3T were reported to be important agents for arsenic removal in contaminated soil [17,18]. Secondary metabolites produced by several *Bacillus* species are environmentally sustainable and can serve as alternatives to chemicals used in various industries and pharmaceutical sectors. They can be employed in the production of biomaterials, such as biofuels and biodegradable bioplastics, which may be a sustainable alternative to traditional petroleum-based products [19].

The annotation of *Bacilli* spp. genomes provides a general overview of biosynthetic gene clusters that are responsible for the production of secondary metabolites with antimicrobial activity [20,21]. These secondary metabolites with antagonistic activities are mostly lipopeptides that are able to span the lipid bilayer of pathogens based on their hydrophilic heads and hydrophobic tail [22]. With the help of antiSMASH v. 7.0.0, the lipopeptide iturin and fengycin were able to be predicted and isolated from *B. velezensis* BA-26, showing antimicrobial properties against *Botrytis cinerea* [23] Another important role of full genome analysis is the determination of resistant genes, which reveals the robustness of strains. In relation to *Bacillus megaterium*, strain STB1 harbors genetic elements responsible for stress resistance, including salt tolerance, and was able to promote the growth of tomato plants in salty conditions [24]. All these genes can be acquired through the horizontal transfer of genes that subsequently distinguishes strains of the same species isolated from the same environment.

As *Bacillus* strains become more diverse, the strains of this genus also differentiate and broaden their classifications. *Bacillus glycinifermentans*, since its discovery via 16S rRNA sequencing, has shown a close relationship to *B. sonorensis* and *B. licheniformis* with such properties as antifungal bacteria and probiotic properties for farm animals [25,26,27]. One example of the unique phenotypic diversity of *B. glycinifermentans* is the thermostable acidic lipase of the strain MK840989 from a petrol pump with an optimum temperature of 90 °C and a pH of 5 [28]. Since the environmental conditions of strains differ, a full genome analysis helps to understand the important phenotypic characteristics that help to determine their biotechnological applications. Additionally, a comparison of their phenotypic correlation with their genotypic characteristics helps rectify the lack of phenotypic characteristics of genes that are genetically present. To isolate these microbes with the ability to resist abiotic stress and able to remediate heavy metals, a good approach is to target the rhizospheric microbes of suffrutescent weed plants with these properties. *Senna occidentalis* is a pantropical medicinal weed that is resistant to drought, remediates heavy metals and is associated with plant growth-promoting bacteria [29,30,31]. Our aim was to isolate and characterize a *Bacillus* strain from *Senna occidentalis* to predict its applications in different biotechnological sectors.

In this study, we report on a novel *B. glycinifermentans* MGMM1 isolated from the rhizosphere of *Senna occidentalis*. Full genome annotation and the identification of its phenotypic characteristics, such as growth properties, enzymatic activities, antagonistic activity, Indole-acetic acid (IAA) production and biocontrol ability against *Forl* ZUM2407, were performed. Based on its unique properties, the strain can be used as a biocontrol agent, and the further study of its genome could be useful for biotechnological applications.

## 2. Materials and Methods

### 2.1. Isolation and 16S rRNA Sequencing Analysis

The bacterial strain, MGMM1 was isolated from the rhizospheric soil of the weed plant *Senna occidentalis*, which is native to West Africa. For this purpose, 1 g of rhizosphere soil was added to a sterile test tube containing 9 mL phosphate-buffered saline (PBS) (140 mM NaCl, 5 mM KH_2_PO_4_, 1 mM NaHCO_3_, pH 7.4). After serial dilution, 0.1 mL aliquot (10^−5^ dilutions) was spread on Luria–Bertani (LB) agar medium (g/L: tryptone, 10 g; yeast extract, 5 g; NaCl, 10 g; agar, 15 g) amended with nystatin (100 µg/mL). Plates were further incubated at 30 ± 1 °C. The grown bacteria were preselected based on their morphological characteristics and purified on LB agar medium. The selected bacterial strains were further identified using the 16S rRNA gene. DNA isolation from bacterial strains was performed using a TRIzol kit (Invitrogen, Carlsbad, CA, USA) according to the manufacturer’s protocol. PCR amplification was performed according to Weisburg et al. [32]. The amplificated fragment was purified from 1.2% gel agarose using a Cleanup Mini kit (Evrogen, Moscow, Russia). The obtained DNA fragment was sent to Evrogen for sequencing. The obtained 16S rRNA sequence chromatogram was blasted on the NCBI database for identification.

### 2.2. Preparation of Bacterial Suspension

The bacterial suspension was prepared from an overnight bacterial culture of *B. glycinifermentans* MGMM1 grown in King’s B medium (g/L: Proteose peptone, 20 Glycerol, 10 mL, K_2_HPO_4_, 1.5, MgSO_4_, 1.5, agar, 15) and were harvested using centrifugation (4000× *g* for 10 min at 4 °C). The bacterial cells were subsequently washed with PBS and further resuspended in the same buffer to a final cell optical density of 0.1 at 595 nm.

### 2.3. Determination of Salt and pH Tolerance of B. glycinifermentans MGMM1

The ability of *B. glycinifermentans* MGMM1 to grow under different salt concentrations and pH ranges was tested in 0.5× LB with different salt concentrations and pH levels. The concentrations of NaCl (1%, 3%, 5%, 7% and 9%) were used to evaluate the salt tolerance of MGMM1. The pH ranging from 4 to 10 (adjusted with either 1 M NaOH or 1 M HCl) was used to evaluate the pH tolerance of *B. glycinifermentans* MGMM1. For this purpose, the cell suspension of MGMM1 was inoculated in three replicates in 96-well culture plates (Costar, Cambridge, MA, USA) containing 0.5× LB medium with the above-mentioned salt concentrations and pH to a final optical density of 0.05 at 595 nm. The cell culture plates were incubated at 30 ± 1 °C for 20 h. The bacterial growth was measured once per 60 min using a spectrophotometer Spectro Star Nano (BMG LabTech, Ortenberg, Germany) at 595 nm. A non-inoculated 0.5× LB medium with the above-mentioned salt concentrations and pH ranges was used as a blank.

### 2.4. The Drought Tolerance Ability of B. glycinifermentans MGMM1

The drought tolerance of MGMM1 was performed according to Michel and Kaufmann [33]. For this purpose, the cell suspension of MGMM1 was inoculated in three replicates in 96-well plates (costar, Washington, DC, USA) containing a fresh 0.5× LB medium supplemented with polyethylene glycol 6000 (PEG-6000) to a final osmotic pressure of −0.05 MPa, −0.25 MPa, −0.50 MPa and −0.80 MPa, respectively. Furthermore, culture plates were incubated in three replicates at 30 ± 1 °C for 12 h. The bacterial growth curve was measured each hour using a spectrophotometer (SpectrostarNano BMG Labtech, Ortenberg, Germany) at 570 nm. For statistical viability, the experiment was repeated twice. The obtained result was evaluated according to Alikhani and Mohamadi [34].

### 2.5. Antagonistic Activity against Phytopathogens

The antagonistic activity of MGMM1 against *F. oxysporum* f. sp. *radicis-lycopersici (Forl)* ZUM2407, *Alternaria alternata*, *F. graminearum* and *F.* spp., was carried out using a dual culture (confrontation) assay method on Sabouraud medium (Merck, Darmstadt, Germany). The cell suspension of MGMM1 obtained in Section 2.1 was used as the inoculum.

### 2.6. Hydrolytic Enzymes Production

#### 2.6.1. Qualification of Hydrolytic Enzymes

The ability of *B. glycinifermentans* MGMM1 to produce amylase, protease, chitinase, and cellulase was tested by plating 2 µL of cell suspension on basal medium agar (g/L: 5.8 g K_2_HPO_4_; 3 g KH_2_PO_4_; 1 g NH_4_SO_4_ 0.2 g MgSO_4_ × 7H_2_O; and 0.5% of C_6_H_12_O_6_) supplemented with 1% starch (Sigma Aldrich, St. Louis, MO, USA), 1% skim milk powder (Sigma-Aldrich, St. Louis, MO, USA), 1% chitin (Sigma Aldrich, St. Louis, MO, USA) and 1% carboxymethyl cellulose (Sigma Aldrich, Darmstadt, Germany), respectively. Lipase and amylase activity were screened as previously reported by Sánchez-Porro et al. [35]. Plates were incubated at 30 °C for up to 5 days. The presence of cellulolytic and chitinolytic enzymatic activities was quantified using floating plates agar with 0.15% Congo Red solution and destained with 1 N NaCl. For amylolytic enzyme activity, plates were floated with 1% iodine ACS reagent (Sigma-Aldrich, Darmstadt, Germany). The observation of halo or clearance zones around close areas surrounding the grown bacterial colonies was considered as enzymatic activities. Lipolytic enzymatic activity was qualified through the presence of salt crystals of free fatty acids released by the enzyme around the grown bacterial colonies.

#### 2.6.2. Quantification of Hydrolytic Enzymes Activity

The quantification of amylase, protease and cellulase activity produced by MGMM1 was performed according to the method of Mondal et al. [36]. For this purpose, MGMM1 was cultivated for 4 days at 37 °C with an agitation of 200 rpm/min in basal medium amended with 1% starch, 1% milk powder and 1% carboxymethyl cellulose, respectively. After incubation, bacterial cultures were centrifugated (at 4 °C with 4000 rpm/min for 10 min), and the obtained supernatants were used as crude enzyme extracts. All experiments were conducted in three replicates and repeated twice. A sterile medium amended with the substrates mentioned above was used as blank solutions. The proteolytic activity was then quantified using azocasein according to the method of Silva et al. [37]. The cellulolytic activity was estimated according to the method of Ghose et al. [38]. The chitinolytic and amylolytic activities were measured using the 3,5-Dinitrosalicylic acid (DNS) method [39,40].

### 2.7. Atmospheric Nitrogen Fixation Ability

The ability of MGMM1 to fix atmospheric nitrogen was qualified based on its ability to grow on nitrogen (N)-free medium [41]. For this purpose, the bacterial suspension of MGMM1 (10 µL) was pipetted onto Jensen’s medium agar (g/L: C_12_H_22_O_11_, 20.0; K_2_HPO_4_, 1.0; MgSO₄, 0.5; NaCl, 0.5; FeSO_4_, 0.10; Na₂MoO₄·2H₂O, 0.005; CaCO_3,_ 2.0; agar, 15) and incubated for up to 4 days at 30 ± 1 °C. The growth of inoculated bacterial strain on nitrogen-free media was considered evidence of N_2_ fixation.

### 2.8. Phytohormones Production

The indole-3 acetic acid (IAA) production by MGMM1 was assayed according to Gorden and Paleg [42] and Glickmann and Dessaux [43]. For this purpose, the cell suspension of MGMM1 was inoculated with a ratio of 1:50 into a conical flask containing 10 mL of LB amended with 1% L-tryptophan and incubated at 30 ± 1 °C for 72 h with constant agitation of 180 rpm. After incubation, cells were harvested using centrifugation (for 10 min at 8000 rpm and 4 °C). A total of 1 mL of the obtained supernatant was mixed with 100 μL of H_3_PO_4_ and 2 mL of Salkowski’s reagent (Sigma-Aldrich, St. Louis, MO, USA). The mixture was further incubated for 30 min in a dark place at 28 °C for color development. The intensity of the developed color was measured at 540 nm using a spectrophotometer Spectro StarNano (BMG LabTech, Ortenberg, Germany). The sterile nutrient broth was maintained as a blank. The amount of IAA produced by MGMM1 was assayed using a standard curve prepared under the same conditions with concentrations of IAA (from 1 to 100 μg/mL).

### 2.9. Biosurfactant Production by B. glycinifermentans MGMM1

Biosurfactants producing the ability of MGMM1 was tested as described elsewhere [44,45,46]. Briefly, 50 μL of cell-free supernatant (passed through a filter diameter of 0.45 µm) was carefully placed on the surface of the parafilm M (Merck, Darmstadt, Germany). The presence of biosurfactants was confirmed via the collapse of the droplets deposited on the parafilm as compared with 10% sodium dodecyl sulfate (SDS) and distilled water, which were used as a positive and negative control, respectively.

### 2.10. Biocontrol Ability of B. glycinifermentans MGMM1

The ability of *B. glycinifermentans* MGMM1 to suppress the development of tomato (*Solanum lycopersicum* L.) plant disease caused by *Forl* ZUM2407 was evaluated under laboratory conditions in pots containing Rockwool presoaked in a mixture of plant nutrient solution ((PNS (g/L): 5.0 mM Ca(NO_3_)_2_ × 4H_2_O, 1.18 g; 5.0 mM KNO_3_, 0.5 g; 2.0 mM MgSO_4_ × 7H_2_O, 0.48 g; pantothenic acid solution, 1.0 mL) and a spore suspension of *Forl* ZUM2407, as described by Afordoanyi et al. [47]. Seeds were inoculated for 15 min in cell suspension of MGMM1 prepared in Section 2.1, sown in pots and incubated in the dark to facilitate germination. After germination, plants were grown for 28 days under 90% light and 16:8 day:night cycles. In each group, 90 plants were evaluated. For statistical analysis, the experiment was carried out in triplicate and repeated twice. The disease index (DI) was estimated using the respective formula:

(1) $$\mathrm{DI}\left(\%\right)=\frac{\left[\left(n_{0}\times 0\right)+\left(n_{1}\times 1\right)+\left(n_{2}\times 2\right)+\left(n_{3}\times 3\right)+\left(n_{4}\times 4\right)\right]}{\begin{array}{c} \left(n_{0}+n_{1}+n_{2}+n_{3}+n_{4}\right) \end{array}}\times 100$$

where *n*_0_, *n*_1_, *n*_2_ and *n*_3_, indicate the number of plants with, respectively, indices 0, 1, 2, 3 and 4.

The statistical evaluation of DI among treated groups was performed with originLab pro-SR1b9.5.1.195 (OriginLab Corp., Northampton, MA, USA). ANOVA one-way and post hoc Tukey’s honestly significant difference test at a *p*-value ≤ 0.05 were used to determine significant differences among treatments.

### 2.11. Genome Sequencing Analysis

#### 2.11.1. DNA Isolation

*Bacillus glycinifermentans* MGMM1 chromosomal DNA was isolated as described above in Section 2.1. The quality and quantity control of the obtained DNA was verified via gel electrophoresis and Qubit 4 Fluorometer (Invitrogen, Carlsbad, CA, USA).

#### 2.11.2. Genome Sequencing and Analysis

The whole genome was sequenced by CeGaT Biotechnology Co., Ltd. (Tübingen, Germany). The assessing read quality scores were obtained using FastQC v. 0.11.2 [48]. Adapters and low-quality reads were trimmed using Trimmomatic v. 0.36. Unicycler v. 0.5.0 [49] and Spades v. 3.12 [50,51] were used for the de novo genome assembly. The obtained contigs from the final assembly data created with Unicycler and scaffolds from Spades were blasted for closed-related genome research. Further, FASTANI [52], ANIb (average nucleotide identity based on BLAST) and the webserver program tool JSpeciesWS (https://jspecies.ribohost.com/jspeciesws/#home, accessed 22 April 2022) were applied to identify the most closely related genome species. SCAR-web (webserver for contigs rearrangements) [53] was further used to rearrange contigs of the target draft genome. GAPPadder v. 1.10 [54] and GapBlaster v. 1.1.1 [55] were used to fill and close gaps between contigs. The genome annotation was performed using the automatic system NCBI Prokaryotic Genome Annotation Pipeline (http://www.ncbi.nlm.nih.gov/genome/annotation_prok, accessed on 4 December 2022). SEED server was used to analyze the subsystem distribution in the strain MGMM1 [56]. The antiSMASH v. 7.0 [57] was used to predict antimicrobial secondary metabolite biosynthetic gene clusters in MGMM1 and compare them with the related genomes of *B. glycinifermentans* obtained from the NCBI database. For this purpose, only the complete and closed genomes of strains (*B. glycinifermentans* KBN06P03352, BGLY and SRCM103574, with accession numbers CP023481.1, NZ_LT603683.1 and CP035232.1, respectively) were used. The Comprehensive Antibiotic Resistance Database (CARD) was used to predict the presence of antimicrobial resistance genes [58].

## 3. Results

A total of 25 strains were purified, and among the identified isolates, *B. glycinifermentans* was selected for further study due to the limited research conducted on this species.

### 3.1. Drought, Salt and pH Tolerance of B. glycinifermentans MGMM1

**Figure 1:**
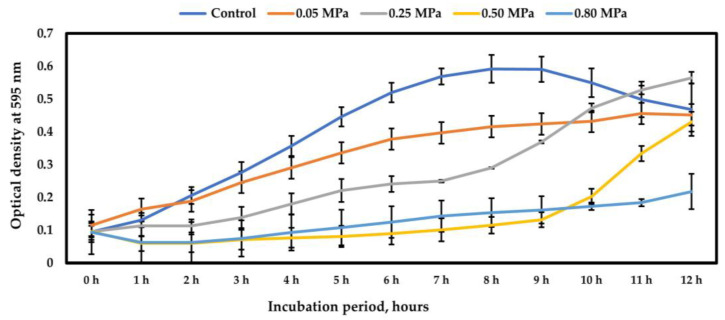
Growth curve of *B. glycinifermentans* MGMM1 under different drought conditions.

The ability of MGMM1 to grow under drought conditions tested using PEG-6000 can be seen in Figure 1. As can be observed, under osmotic pressure ranging from −0.05 to −0.25 MPa, the growth (exponential phase) of MGMM1 occurred immediately after incubation. In addition, under these conditions, the linear growth phase of MGMM1 continued throughout the entire incubation time, unlike the control, in which the stationary phase occurred after 9 h. However, under the osmotic pressure of −0.5 MPa, an increment in the log growth phase of MGMM1 was observed. The exponential phase of MGMM1 occurred after 9 h of incubation. A similar result was obtained when *B. glycinifermentans* MGMM1 was incubated under the osmotic conditions of −0.8 MPa. In this case, the exponential growth phase of MGMM1 was recorded 11 h after incubation. The obtained result shows that *B. glycinifermentans* MGMM1 is a drought-tolerant bacterium.

**Figure 2:**
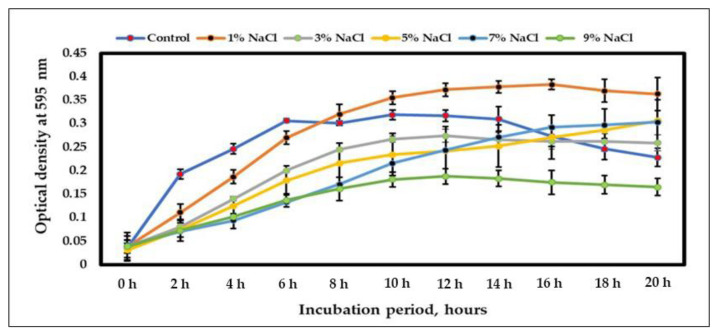
The growth curve of *B. glycinifermentans* MGMM1 under different levels of salinity (NaCl).

To evaluate the ability of *B. glycinifermentans* MGMM1 to grow under different salt concentrations, the growth rate of the strain was assayed in nutrient broth containing various NaCl concentrations ranging from 1 to 9%. The obtained result revealed that MGMM1 is able to grow both without salt and under saline conditions of up to 9% NaCl (Figure 2). This indicated that MGMM1 quickly adapted to salty conditions and rapidly started growing into the exponential phase. In addition, the log phase of MGMM1 at 7% NaCl remained up to 10 h. Moreover, it was found that a salt concentration of more than 9% NaCl inhibited the growth rate of MGMM1, as compared with other salt concentrations tested. The exponential growth phase of *B. glycinifermentans* MGMM1 at 9% NaCl lasted up to 12 h after incubation. The general cell density of MGMM1 was less than 0.2 at 595 nm.

**Figure 3:**
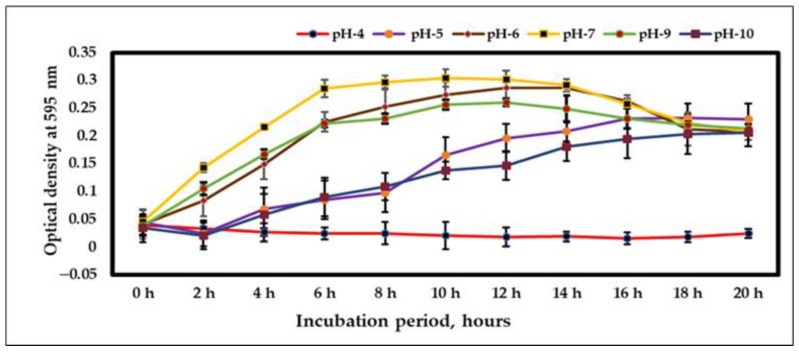
Growth curve of *B. glycinifermentans* MGMM1 under different pH conditions.

To investigate the effect of different pH conditions on the growth of MGMM1, the bacterial culture was incubated at different pH ranging from 4 to 9 (Figure 3). The result revealed that in acidic conditions with a pH of 5, the growth (exponential) phase of *B. glycinifermentans* MGMM1 occurred 2 h after incubation. The linear growth phase of MGMM1 remained up to 16 h. However, under pH conditions below 5, the growth of MGMM1 was inhibited. The cell density of the incubated strain was lower than the initial optical density (from 0.05 to 0.024). Under pH conditions ranging between 6 and 9, the exponential growth phase of MGMM1 was observed immediately after incubation with a stationary phase occurring 6 to 14 h after incubation. Moreover, under alkaline conditions with a pH of 10, the growth ability of MGMM1 was almost similar to those registered at pH 5. In this case, the exponential growth phase occurred after 2 h, and the linear growth phase remained present throughout the entire incubation time (up to 20 h). The obtained result demonstrated that MGMM1 is a neutrophile bacterial strain with optimal pH growth conditions of 5 to 9.

### 3.2. Antagonistic Activity against Phytopathogens

**Figure 4:**
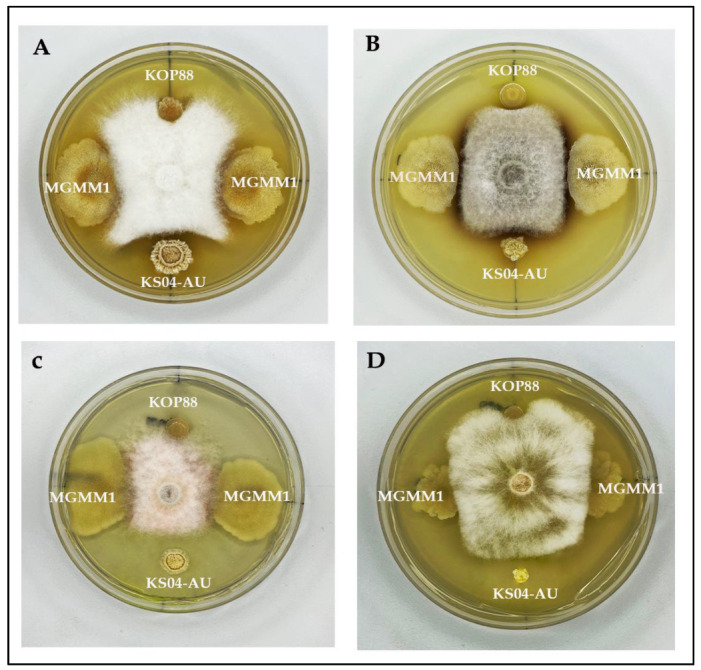
Antagonistic activity of *B. glycinifermentans* MGMM1 against *Forl* ZUM2407 (**A**), *A. alternata* (**B**), *F. graminearum* (**C**) and *F.* spp. (**D**). *Bacillus velezensis* KS04AU [59] and *Pseudomonas* spp. KOP88 were used as positive and negative controls, respectively. Plates were incubated for 6 days at 27 ± 1 °C.

To screen the potential of *B. glycinifermentans* MGMM1 to inhibit the growth of plant pathogens, its antagonistic activity was evaluated. After incubation, the result showed that *B. glycinifermentans* MGMM1 inhibited the growth of all phytopathogenic fungi used in this study (Figure 4). Among these tested pathogens, a significant zone of inhibition was observed against *F.* spp., *F. graminearum*, *A. alternata and Forl* ZUM2407. The most effective antagonistic activity was observed against *Forl* ZUM2407 and *F. graminearum* whilst the lowest was against *A. alternata*. The obtained result indicated that *B. glycinifermentans* MGMM1 exhibited a broad spectrum against these phytopathogenic fungi.

### 3.3. Hydrolytic Enzymes Production

**Figure 5:**
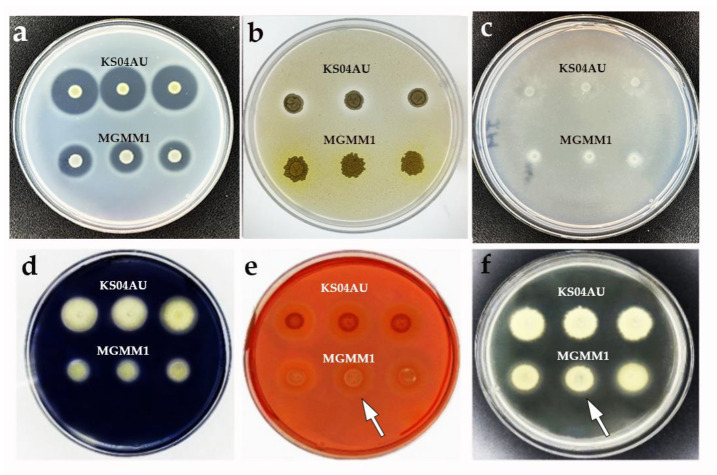
Atmospheric nitrogen fixation and hydrolytic enzymes producing ability of *B. glycinifermentans* MGMM1. Picture showing the (**a**) protease, (**b**) phytase, (**c**) atmospheric nitrogen fixation ability, (**d**) amylase, (**e**) cellulase and (**f**) lipase activity of MGMM1. Arrows indicate the formation of halo zones and crystals surrounding the growing bacterial colonies. *Bacillus velenzesis* KS04AU was used as a positive control.

The ability of MGMM1 to produce hydrolytic enzymes on minimal media supplemented with their corresponding substrates as a carbon source revealed the presence of proteolytic, amylolytic, lipolytic and cellulolytic enzymes (Figure 5).

The evidence of these enzymes was observed through the formation of zones surrounding the growing bacterial colonies. However, the activities of phosphohydrolytic enzymes were not detected since no halo zones were observed around the growing bacterial colonies on phytic agar plates (Figure 5b). The ability of MGMM1 to fix atmospheric nitrogen was also assayed based on its ability to grow on a nitrogen-free medium. The results showed that MGMM1 is able to use atmospheric nitrogen gas to synthesize cellular protein necessary for cell growth (Figure 5c). Furthermore, the activities of hydrolytic enzymes (amylase, protease and cellulase) were quantified (Table 1). The amounts of protease, amylase and cellulase enzymatic activities produced by MGMM1 were measured as 4.82 ± 1.04, 0.84 ± 0.05 and 0.35 ± 0.02, respectively. A similar result was obtained in terms of IAA production. The amount of IAA produced by MGMM1 in the presence of tryptophan was quantified as 48.96 ± 1.433 μg/mL.

### 3.4. Biosurfactant Production

**Figure 6:**
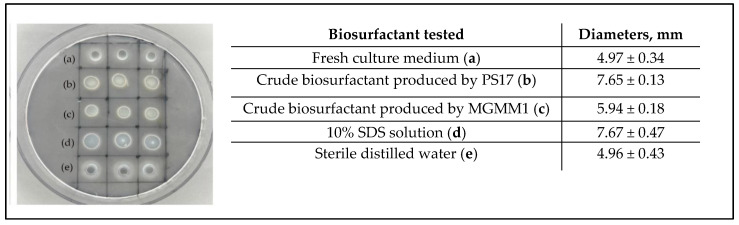
Screening preliminary test of the biosurfactant producing-ability of *B. glycinifermentans* MGMM1 visualized on a strip of parafilm M. Fresh culture medium (a), crude biosurfactant produced by *B. mojavensis* PS17 (b), crude biosurfactant produced by MGMM1 (c), 10% SDS solution (d) and sterile distilled water (e).

The confirmation of the biosurfactant-producing ability of MGMM1 was carried out using a preliminary screening test on parafilm M. The obtained result showed that MGMM1 produces biosurfactants since the droplet diameter of cell-free supernatant obtained from MGMM1 was larger than those of negative controls (Figure 6).

### 3.5. Biocontrol Proprieties of B. glycinifermentans MGMM1

**Figure 7:**
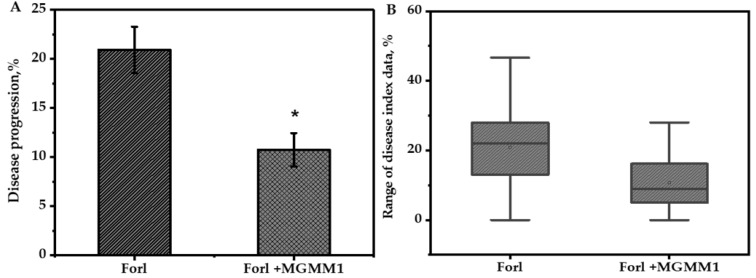
Biocontrol properties of *B. glycinifermentans* MGMM1 in the suppression of disease development of tomato foot and root induced by *Forl* ZUM2407. (**A**)—disease index of treated group (data are represented as mean ± SD). (**B**)—range of disease development data of tomato plant. (*)—significant difference among groups at *p*-value ≤ 0.05.

To confirm the ability of the strain MGMM1 in planta to inhibit the growth of tomato plant diseases caused by *Forl* ZUM2407, its biocontrol properties were evaluated (Figure 7). Twenty-five days after sowing, a statistical (*p* ≤ 0.05) inhibition of disease development was observed in the group of plants pretreated with *B. glycinifermentans* MGMM1, as compared to control plants with only *Forl* ZUMM2407. The disease index in the group of plants without treatment was almost 1.94 times higher than those pretreated with *B. glycinifermentans* MGMM1. Their DI values were evaluated as 20.91 ± 2.36% and 10.76 ± 1.69%, respectively. Thus, in this study, the obtained result indicated the biocontrol ability of *B. glycinifermentans* MGMM1 to inhibit up to 51.45 ± 8.08% of tomato foot and root disease induced by *Forl* ZUM2407.

### 3.6. Genome Assembly and Analysis

The average nucleotide identity (ANI) analysis using the JSpecies server showed MGMM1 to be closely related to *B. glycinifermentans* SRCM103574 (accession number CP035232.1). The prokaryotic genome automatic annotation pipeline (PGAP) of MGMM1 revealed 4259 putative coding sequences (CDS) with an encoding density of 95.75% attributed to biological functions and 4.25% considered hypothetical genes (genes with unknown functions). The genome contains 4,346,570 bp with a total percentage of 46.3% G + C content. The genome carried 73 Genes (RNA), 3 complete rRNAs, 65 tRNAs, and 5 ncRNAs. The whole genome sequence of MGMM1 was deposited in the NCBI database under Bioproject with the accession number PRJNA825337.

**Figure 8:**
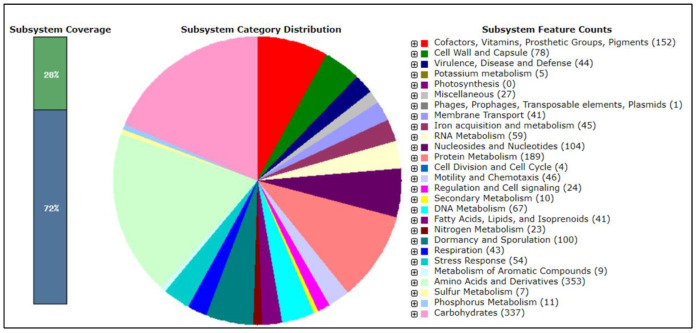
Subsystem Information for *B. glycinifermentans* MGMM1. In subsystem coverage, 28% is indicated with a total of 1311 genes (1252 non-hypotheticals and 59 hypotheticals) and 72% is not indicated in subsystem coverage with a total of 3460 genes (1657 non-hypotheticals and 1803 hypotheticals).

Since the results demonstrated that MGMM1 is a potential biocontrol strain (Section 2.8), we focused on genes and cluster genes which may be involved in the biosynthesis of compounds with antimicrobial properties, as well as those that trigger resistance responses to phytopathogens in plants. The genome analysis using NCBI annotation revealed the presence of several genes involved in plant growth promotion (acetolactate synthase, *als*S), genes for phosphate solubilization (*pst*A, *pst*B and *pst*C), nitrogen fixation (*Nif*U) and genes involved in the resistance to the heavy metal antimony (*ars*B and *ars*C). The subsystem category distribution in MGMM1 genome using RAST (Figure 8) revealed the presence of genes involved in the regulation system of bacitracin stress responses and resistance to antibiotics and toxic compounds, such as streptothricin resistance, copper homeostasis, cobalt-zinc-cadmium resistance, fluoroquinolones and fosfomycin resistance, beta-lactamase and several multidrug-resistance efflux pumps.

**Figure 9:**
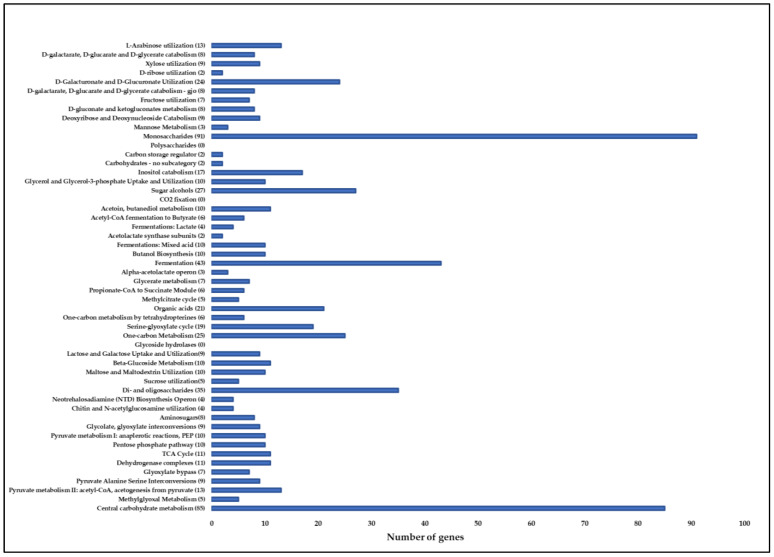
Genes involved in carbohydrate metabolism in *B. glycinifermentans* MGMM1.

Genes encoding phosphate transporters; nitrogen metabolisms such as nitric oxide synthase, nitrate and nitrite ammonification genes were predicted in the MGMM1 genome. Several genes related to siderophore biosynthesis (such as bacillibactin siderophore and siderophore anthrachelin) that are involved in iron chelating ability were found in MGMM1. Genes responsible for the anaerobic degradation of aromatic compounds such as the hydroxyaromatic decarboxylase genes family were predicted in MGMM1. In addition, genes involved in central carbohydrate metabolism and amino-sugars, such as chitin, N-acetylglucosamine utilization and neotrehalosadiamine (NTD) biosynthesis, were predicted. The genes involved in beta-glucoside metabolism, butanol biosynthesis, glycerol and glycerol-3-phosphate uptake and utilization were also predicted in the genome of MGMM1. In contrast, no glycoside hydrolase genes were found in MGMM1 (Figure 9).

The comparative analysis of the secondary metabolisms of *B. glycinifermentans* MGMM1 with related strains using antiSMASH v.7.0 is shown in Table 2. The result revealed that MGMM1 and KBN06P03352 possess, respectively, ten putative gene clusters responsible for the biosynthesis of antimicrobial metabolite, while BGLY and SRCM103574 harbor nine regions. Two putative gene cluster types, polyketide synthase type III (T3PKS) and the lassopeptide/RRE-containing type predicted in MGMM1 and KBN06P03352, did not match the most well-known biosynthetic gene cluster. BGLY and SRCM103574 also display two regions (cluster types: terpene and T3PKS) in their genomes, which did not match any of the biosynthetic gene clusters from the repository of known biosynthetic gene clusters. Moreover, a core of five putative gene clusters with a BLAST hit similarity to the most well-known biosynthesis genes clusters of Bacillibactin/bacillibactin E/bacillibactin F, Lichenysin, Schizokinen, Fengycin and Butirosin A/butirosin B was predicted in all four genomes. The biosynthetic genes cluster responsible for the synthesis of plipastatin, present in MGMM1, was not predicted in KBN06P03352, BGLY and SRCM103574. Regions with BLAST hit similarity to Bacitracin and Sporulation killing factor biosynthetic gene clusters were predicted only in BGLY and SRCM103574 genomes. The tyrocidine biosynthetic gene cluster predicted in KBN06P03352 was absent in the genomes of MGMM1, BGLY and SRCM103574

The antibiotic resistome analysis of MGMM1 using the Comprehensive Antibiotic Resistance Database (CARD) is shown in Table 3. The antimicrobial resistance (AMR) revealed that *B. glycinifermentans* MGMM1 carried an antibiotic resistance ontology (ARO) term belonging to the fosfomycin thiol transferase (FosBx1) AMR family, which encodes the phosphonic acid antibiotic resistance proteins with antibiotic inactivation resistance mechanism. Several *van*T, *van*Y and *van*W AMR gene families encode the glycopeptide antibiotic resistance proteins, acting through the resistance mechanism of antibiotic target alteration. The class A *Bacillus cereus* Bc beta-lactamase ARM gene family involved in the resistance of cephalosporin and the unsaturated beta-lactam antibiotics drug class was predicted in the genome of MGMM1. A broad specificity small multidrug resistance (SMR) antibiotic efflux pump belonging to the qacJ ARO term, which provides the bacteria resistance against disinfecting and antiseptic agent class drugs were predicted in MGMM1. The obtained result is consistent with those obtained using RAST server.

## 4. Discussion

The search for new strains for the biological control of diseases and that also display plant growth stimulation activity is very important for the gradual change from harmful agrochemicals to biopreparations. In the search for these strains, *B. glycinifermentans*, in comparison to the closely related species *B. licheniformis*, has been less characterized based on phenotypic properties. Genotypically, there is plenty of information on their full genome assembly, which includes the strain GO-13^T^ (= KACC 18425^T ^, = NRRL B-65291^T^) [25] and was first assigned the name *B. licheniformis* based on 16S rRNA sequencing; the strain B-27 [60], using “Single Molecule, Real-Time” (SMRT) sequencing; and the strain JRCGR-1 [61], using an Illumina NextSeq 500 platform. Based on its phenotypic characteristics, *B. glycinifermentans* has been researched for use in different sectors, from anticancer metabolites to probiotics [62,63]. To determine the application of *B. glycinifermentans* MGMM1, the phenotypic properties revealed its application potential for plant protection, whilst based on its genotypic characteristics, it could be utilized as a remedial bacterium if further researched.

Regarding its use as a drought inoculant for plants, *B. glycinifermentans* MGMM1 was able to grow in osmotic pressure up to −0.5 MPa. Although in 0.80 MPa, the lag phase was long, MGMM1 showed exponential growth after 12 h, attesting to its drought-resistant ability. A correlation between IAA production and the ability to survive in high osmotic pressure does help plants in drought conditions. Similar results were reported in a recent paper published by Saxena et al. [64], where two strains of *B. glycinifermentans* PN2K3 and PN314 were isolated from *Azadirachta indica* (neem) gum, where the osmotic pressure is high. In addition, its ability to grow in high salt content of 9% and a pH of 10 makes it a suitable candidate for its application in salty soil, as the average pH of seawater is in the range of 7.5–8.5. To attest to our results, the tolerance of *B. glycinifermentans* JRCGR-1 to grow in the basic medium and high salt was 8% (w/v) NaCl and pH 10, respectively [25]. In terms of antifungal activity, *B. glycinifermentans* MGMM1 was able to inhibit all four phytopathogenic fungi. Although the inhibition was not as effective as the positive control (*Bacillus velezensis* KS04AU [59]), *B. glycinifermentans* MGMM1 was still able to inhibit the disease progression of *Forl* ZUM2407 on tomato plants, confirming its usefulness in plant protection. Additionally, due to the high cellulolytic and lipase activity of MGMM1, it can help to degrade plant residue in the soil as a source of carbon for its survival in the plant rhizosphere. Despite the lack of phytase enzymatic activity, the presence of gene-encored 3-phytase and some phosphatases and pyrophosphatases were found in the genome of MGMM1, implying the reduced down expression or no expression of the genes.

The full genome analysis of *B. glycinifermentans* MGMM1 shows the various genes responsible for resistance to heavy metals, such as cobalt and cadmium, as well as antiseptics and several antibiotics. As can be observed in Table 2, *B. glycinifermentans* MGMM1 harbors genes that are resistant to different classes of antibiotics, disinfectants and antiseptic agents, which normally end up in sewage and, subsequently, in farmland [65,66]. This may be a good indicator for its application in sewage treatments, as it can withstand these harsh conditions. Although there is no research on its application for bioremediation, an article by Li et al. [67] compared the genome of *Bacillus* sp. S3 to other *Bacillus* species to determine its resistance to heavy metal antimony (Sb(III)-related genes). The authors were able to determine that the G + C content between genes (*ars*B 1,2,3 and *arsC*) of *Bacillus* sp. S3 and other selected species were key factors for heavy metal resistance. In our case, *B. glycinifermentans* MGMM1 harbors the genes *ars*B and *ars*C but not the unique *aio*B gene of S3. In relation to *B. glycinifermentans*, according to our research, there have been no studies published on the bioremediation of heavy metals but more on the closely related *B. licheniformis* [25]. Based on the different mechanisms of heavy metal bioremediation, an extracellular polymeric substance of *B. licheniformis* KX657843 was able to absorb Cu(II) and Zn(II) by approximately 80% [68]. Another interesting finding is that *B. licheniformis* A6 was able to grow in high concentrations of As, Cr, Hg, Mn, Se, Pb, Co, Cd and Zn with the ability to oxidize arsenite into arsenate within 2–4 days [69]. The above statements provide the basis for a focus on the research and practical application of MGMM1 (resistance genes for Cu homeostasis, Co–Zn–Cd resistance) in wastewater treatment, since its harbors the *ars*B and *ars*C genes.

The increase in the resistant of microbes to the most potent antibiotics has directed researchers’ interest toward biosurfactants as pharmaceutical drugs, due to their ability to inhibit most drug-resistant bacteria as well as their anticancer and antitumor activity [70,71]. In terms of secondary metabolites gene clusters, the anionic cyclic lipoheptapeptide biosurfactant (lichenysin), produced by *B. licheniformis* [72] and also *B. glycinifermentans* [25], was also found in the genome of MGMM1. The genome also contains the catechol-based siderophore bacillibactin, which is involved in the chelating of ferric ions [73]. The lipopeptide biosurfactant has also been reported to have a broad spectrum of inhibitory activity against phytopathogens and virulent drug-resistant bacteria [74,75]. Out of all the four complete genome assemblies on the NCBI database, MGMM1 revealed a cluster for the production of the cationic lipopeptide laterocidine, which is produced by *Brevibacillus* species and has a bacteriocidal effect against the antibiotic-resistant pathogens of the “ESKAPE” group [76]. Another common siderophore, schizokinen, is found in all four compared genomes of *B. glycinifermentans*, which has been reported to be a potent iron-chelating siderophore produced by cyanobacteria [77]. The presence of these genes does not directly correlate to their expression, which can be observed through the presence of the phytase gene but is phenotypically absent. This provides further information for the future research on the other applications of *B. glycinifermentans* MGMM1 proposed in this study.

## 5. Conclusions

The search for new potential *Bacillus* sp. with the properties to protect plants and animals is important for the reduction in agrochemical and antibiotic use in agriculture. This can be achieved due to their phenotypic characteristics, based on their genomic conformation. Taking into consideration the increase in heavy metal and xenobiotics contaminants in the environment, an overview of the genome on different databases helps to predict their applications. Here, we characterized the bacterium, *B. glycinifermentans*, MGMM1 isolated from the plant *Senna occidentalis* and confirmed its application as a biocontrol agent against phytopathogens based on its phenotypic characteristics. Furthermore, full genome analysis showed its resistance to different classes of antibiotics and some heavy metals. An antiSMASH annotation of the secondary metabolite cluster genes presented biosurfactants that could be further isolated and researched for their use as antibiotics. We, therefore, propose *B. glycinifermentans* MGMM1 as a good candidate for plant protection with further research on the strain required, regarding its application in other sectors. Likewise, an interesting piece of information concerns the presence of the Nonribosomal peptides, type I polyketide synthase, and terpenes cluster genes responsible for the synthesis of laterocidine, reported to inhibit the “ESKAPE” group of drug-resistant pathogens. In general, we suggest that *B. glycinifermentans* MGMM1 is a good candidate for controlling diseases caused by phytopathogens and as plant growth-promoting bacteria. Further research on its phenotypic characteristics for pharmaceutical and bioremediation applications will be carried out by isolating the predicted lipopeptides against potential pathogens and researching its ability to immobilize or sequester heavy metals.

## Figures and Tables

**Table 1:** Quantitative analysis of the hydrolytic enzyme produced by *B. glycinifermentans* MGMM1.

Quantitative Analysis	
Hydrolytic Enzymes, U/mL	
Protease	4.82 ± 1.04
Amylase	0.84 ± 0.05
Cellulase	0.35 ± 0.02
Phytohormones, μg/mL	
Indole-3-acetic Acid	48.96 ± 1.43

**Table 2:** Analysis of the secondary metabolisms of *B. glycinifermentans* MGMM1 and three related strains.

Region	Metabolite Type	Most Similar Known Cluster	Similarity (%)
*B. glycinifermantans* MGMM1			
1	NRPS	plipastatin	38
2	betalactone	Fengycin	53
3	NRPS, T1PKS, terpene	Laterocidine	5
4	T3PKS	-	-
5	NRP-metallophore, NRPS	bacillibactin/bacillibactin E/bacillibactin F	100
6	lassopeptide, RRE-containing	-	-
7	lanthipeptide-class-ii	geobacillin II	50
8	NRPS	lichenysin	100
9	thiopeptide	butirosin A/butirosin B	7
10	NI-siderophore	schizokinen	60
*B. glycinifermentans* KBN06P03352			
1	NRPS	fengycin	26
2	betalactone	fengycin	53
3	NRPS, T1PKS, terpene	tyrocidine	12
4	T3PKS	-	-
5	NRPS	bacillibactin/bacillibactin E/bacillibactin F	100
6	lassopeptide, RRE-containing	-	-
7	lanthipeptide-class-ii	geobacillin II	50
8	NRPS	lichenysin	100
9	thiopeptide	butirosin A/butirosin B	7
10	NI-siderophore	schizokinen	60
	*B. glycinifermentans* BGLY		
1	sactipeptide	sporulation killing factor	85
2	NRPS	lichenysin	100
3	thiopeptide	butirosin A/butirosin B	7
4	NI-siderophore	schizokinen	60
5	betalactone	fengycin	53
6	terpene	-	-
7	T3PKS	-	-
8	NRPS	bacitracin	88
9	NRP-metallophore, NRPS	bacillibactin/bacillibactin E/bacillibactin F	100
*B. glycinifermentans* SRCM103574			
1	sactipeptide	sporulation killing factor	85
2	NRPS	lichenysin	100
3	thiopeptide	butirosin A/butirosin B	7
4	NI-siderophore	schizokinen	60
5	betalactone	fengycin	53
6	terpene	-	-
7	T3PKS	-	-
8	NRPS	bacitracin	88
9	NRP-metallophore, NRPS	bacillibactin/bacillibactin E/bacillibactin F	100

N.B. NRPS, non-ribosomal peptide synthetase; T1PKS and T3PKS, type I and III PKS (polyketide synthase), respectively; NI-siderophore, NRPS-independent IucA/IucC-like siderophores.

**Table 3:** Antimicrobial resistance (AMR) gene family predicted in *B. glycinifermentans* MGMM1.

ARO Term	AMR Gene Family	Drug Class	Resistance Mechanism
FosBx1	fosfomycin thiol transferase	phosphonic acid antibiotic	antibiotic inactivation
vanT gene in vanG cluster	glycopeptide resistance gene cluster, vanT	glycopeptide antibiotic	antibiotic target alteration
qacJ	small multidrug resistance (SMR) antibiotic efflux pump	disinfecting agents and antiseptics	antibiotic efflux
qacJ	small multidrug resistance (SMR) antibiotic efflux pump	disinfecting agents and antiseptics	antibiotic efflux
vanY gene in vanB cluster	vanY, glycopeptide resistance gene cluster	glycopeptide antibiotic	antibiotic target alteration
BcIII	class A Bacillus cereus Bc beta-lactamase	cephalosporin, penem	antibiotic inactivation
vanT gene in vanG cluster	glycopeptide resistance gene cluster, vanT	glycopeptide antibiotic	antibiotic target alteration
qacJ	small multidrug resistance (SMR) antibiotic efflux pump	disinfecting agents and antiseptics	antibiotic efflux
vanW gene in vanI cluster	vanW, glycopeptide resistance gene cluster	glycopeptide antibiotic	antibiotic target alteration

## Data Availability

Not applicable.
